# Inverse design of compact nonvolatile reconfigurable silicon photonic devices with phase-change materials

**DOI:** 10.1515/nanoph-2023-0637

**Published:** 2024-01-15

**Authors:** Maoliang Wei, Xiaobin Lin, Kai Xu, Yingchun Wu, Chi Wang, Zijia Wang, Kunhao Lei, Kangjian Bao, Junying Li, Lan Li, Erping Li, Hongtao Lin

**Affiliations:** The State Key Lab of Brain-Machine Intelligence, Key Laboratory of Micro-Nano Electronics and Smart System of Zhejiang Province, College of Information Science and Electronic Engineering, Zhejiang University, Hangzhou 310027, China; Key Laboratory of 3D Micro/Nano Fabrication and Characterization of Zhejiang Province, School of Engineering, Westlake University, Hangzhou, Zhejiang 310030, China; Institute of Advanced Technology, Westlake Institute for Advanced Study, Hangzhou, Zhejiang 310024, China; Hangzhou Institute for Advanced Study, University of Chinese Academy of Sciences, Hangzhou 310024, China

**Keywords:** adjoint method, robust inverse design, phase change material, silicon photonics

## Abstract

In the development of silicon photonics, the continued downsizing of photonic integrated circuits will further increase the integration density, which augments the functionality of photonic chips. Compared with the traditional design method, inverse design presents a novel approach for achieving compact photonic devices. However, achieving compact, reconfigurable photonic devices with the inverse design that employs the traditional modulation method exemplified by the thermo-optic effect poses a significant challenge due to the weak modulation capability. Low-loss phase change materials (PCMs) exemplified by Sb_2_Se_3_ are a promising candidate for solving this problem benefiting from their high refractive index contrast. In this work, we first developed a robust inverse design method to realize reconfigurable silicon and phase-change materials hybrid photonic devices including mode converter and optical switch. The mode converter exhibits a broadband operation of >100 nm. The optical switch shows an extinction ratio of >25 dB and a multilevel switching of 41 (>5 bits) by simply changing the crystallinity of Sb_2_Se_3_. Here, we experimentally demonstrated a Sb_2_Se_3_/Si hybrid integrated optical switch for the first time, wherein routing can be switched by the phase transition of the whole Sb_2_Se_3_. Our work provides an effective solution for the design of photonic devices that is insensitive to fabrication errors, thereby paving the way for high integration density in future photonic chips.

## 1 Introduction

Silicon photonics, given its seamless integration with complementary metal oxide semiconductor (CMOS) technology, has gained significant traction and has found applications in diverse domains such as optical computing [1], microwave photonics [2], and optical communications [3]. Similar to Moore’s Law [4], downsizing optical devices can enhance chip integration density, paving the way for augmented functionalities. However, conventional design methods primarily hinge on established photonic design libraries, leveraging standard structural units. These conventional approaches are typically constrained by a limited set of adjustable parameters, which curtails both the performance optimization and size reduction of devices. In stark contrast, the inverse design, enabled by the algorithm, has recently emerged as a promising avenue for the meticulous design and optimization of photonic devices [5], including wavelength division multiplexers (WDM) [6], [7], mode converters (MC) [8], [9], and particle accelerators [10]. Moreover, the strategic infusion of perturbations during the optimization phase ensures the creation of robust, compact photonic devices [11]. Thus, inverse design stands out as a formidable approach to propel optical chip integration.

Despite the commendable advancements inverse design has brought to passive photonic devices, there is still uncharted territory waiting to be explored in the realm of reconfigurable devices. While there have been documented instances of reconfigurable integrated photonic devices that harness inverse design principles (notably those controlled using metal heaters), the scale of these devices remains notably large [12]. This can be largely attributed to the subtle shifts in the refractive index induced by the thermo-optic effects [13]. Chalcogenide phase change material (PCM) with self-sustaining high refractive contrast (typically Δ*n* > 0.5) [14], are promising candidates for achieving compact light manipulation devices [15]. Moreover, the ultra-low loss PCMs maintain low optical absorption at 1550 nm during the phase transition [16], [17], which enables the nonvolatile pure-phase modulation. PCMs have found extensive applications in reconfigurable optical devices and networks, encompassing photonic attenuators [18], [19], [20], [21], optical filters [22], optical phase shifters [23], [24], [25], [26], optical convolutional kernel [27], photonic memory array [28], [29], neuromorphic computing networks [30], and optical tensor core [31], [32]. Therefore, PCM that heralds significant refractive index modulation is a promising solution for the design of compact, reconfigurable, inverse-designed photonic devices, and a series of devices have been demonstrated including optical switches [33], [34], [35], MCs [36], [37], and WDMs [38]. Nonetheless, a pressing concern remains: the predominant reliance of these devices on the pixel-level manipulation of PCMs. This leads to a dependency on spatial light modulation systems, which, regrettably, are not suitable for large-scale integration, and the performance and advantages of reconfigurable devices have not been fully leveraged yet.

In this study, we developed a robust inverse design method for designing reconfigurable photonic devices. Several thermally inducible Sb_2_Se_3_-based photonics devices were demonstrated, including reconfigurable MC and reconfigurable optical switch (OS), and experimentally demonstrated crystallization-induced routing switching of fabrication error-tolerant OS. The designed MC possesses a low-loss operation bandwidth of >100 nm (<1 dB). The designed OS exhibits a low insertion loss (IL) (<0.45 dB) with a high extinction ratio (ER) (>25 dB) at 1563.5 nm. Moreover, the OS exhibits a multilevel switching induced by the crystallization of the Sb_2_Se_3_ structure of 41 levels (>5 bits) in our design. To the best of our knowledge, for the first time, an OS that is switched induced by the phase transition of the whole Sb_2_Se_3_ structure was experimentally demonstrated. The fabricated OS exhibits an insertion loss of <1.3 dB (<5.0 dB) and an extinction ratio of >8.5 dB (>14 dB) at 1550 nm, corresponding to the amorphous (crystalline) state. Our study has demonstrated the feasibility of compact and reconfigurable photonic devices exhibiting insensitivity to fabrication error, paving the way for improving the integration density of integrated photonics chips.

## 2 Results and discussion

### 2.1 Design principle

**Figure 1:**
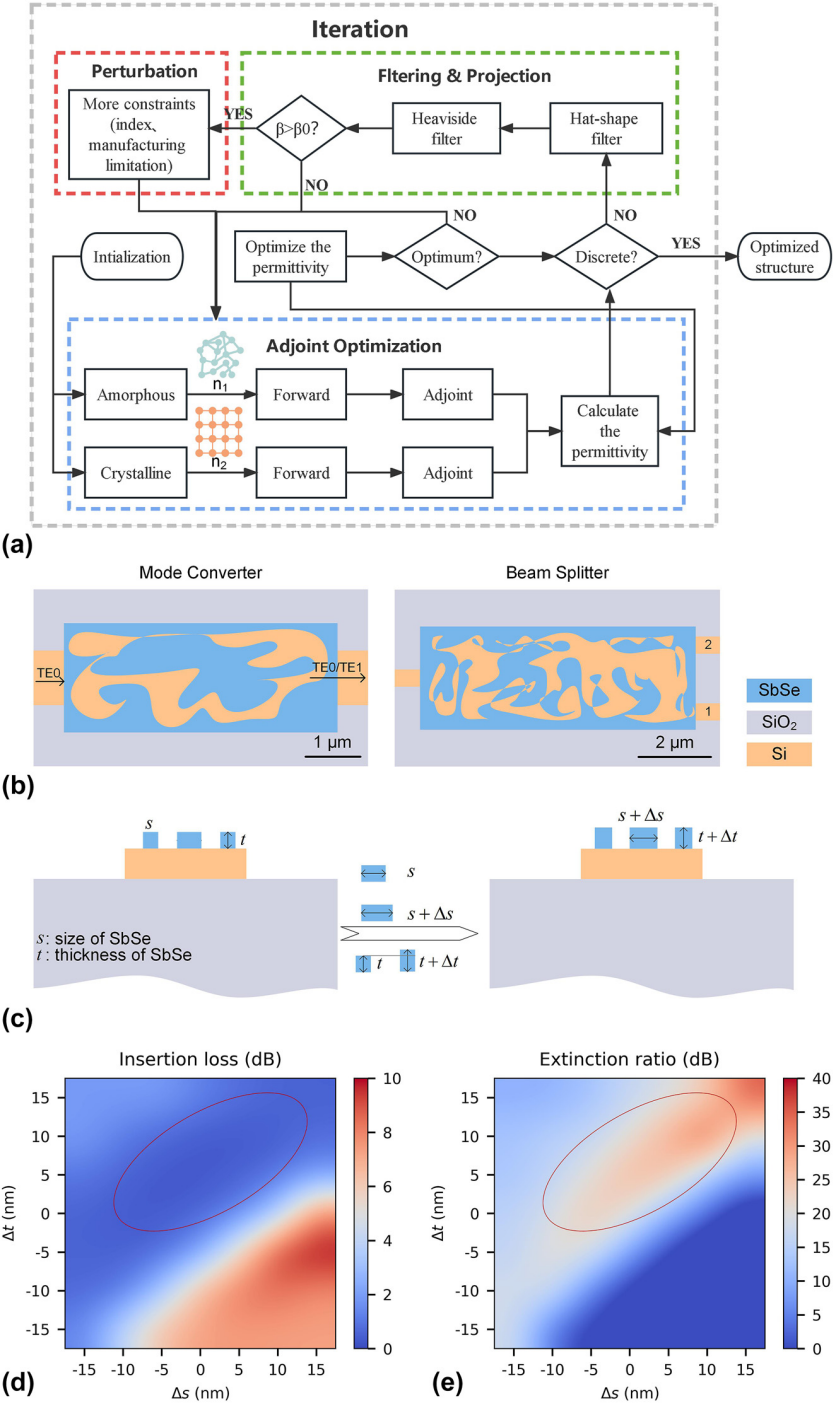
The algorithm architecture of robust inverse design and the robustness analysis of an inversed-designed photonic device. (a) The flow chart of the robust inverse design algorithm. (b) The structural diagram of the MC and OS. (c) The schematic diagram illustrates the dimensional and thickness variations of the Sb_2_Se_3_ structure. The IL (d) and ER (e) of the OS, with Sb_2_Se_3_ in its amorphous state, were simulated for various sizes (Δ*s*) and thickness (Δ*t*) of the Sb_2_Se_3_ pattern. The prominently marked red ellipsoid indicates that OS still exhibits low IL and a high ER at certain fabrication errors.

The refractive index dominant reconfigurable photonic devices can be achieved by employing a robust inverse design algorithm (refer to SI.1 for comprehensive details). The algorithm flow chart of the robust inverse design is depicted in Figure 1(a). The algorithm consists of two judgment units (optimum and discrete) and three parts (adjoint optimization, filter and projection, and perturbation). The judgment units are employed to determine whether to continue optimization or not. The adjoint optimization contains a parallel optimization of permittivity, making sure the geometry of different indices is the same. This design scheme offers a convenient way for reconfiguring the function of photonic devices, which is configuring the desired function by simply changing the refractive index of the functional material. The filtering and projection part is used for the binarization of the permittivity, achieving a fabricable design. The perturbation is adopted to consider manufacturing errors (size and thickness of functional material) to achieve robust design. Employing this algorithm, a refractive index inducible reconfigurable photonic device with fabrication error tolerance can be designed.

To achieve compact reconfigurable photonic devices, a hybrid integration of Sb_2_Se_3_ and silicon was adopted to achieve efficient refractive index configuration. To achieve a higher refractive index modulation, a thicker Sb_2_Se_3_ was adopted. Here, the thickness of Sb_2_Se_3_ and Si is 120 nm and 220 nm, respectively. The schematic layout of the reconfigurable MC and reconfigurable OS designed by robust inverse design are shown in Figure 1(b). To achieve manufacturable devices with low insertion loss and high extinction ratio, the footprint of MC and OS is chosen to be 5 μm × 2 μm and 8 μm × 3 μm, respectively. During the iteration, the geometry of Sb_2_Se_3_ on top of silicon was optimized for achieving the desired function. The mesh grid of each simulation in the optimization is 20 nm. The refractive index of amorphous (crystalline) Sb_2_Se_3_ is 3.327 (4.150) at 1550 nm, which is the same value as our previous work [39]. To account for the reduction in thickness of Sb_2_Se_3_, the refractive index of crystalline state is set to be 3.8 in simulation, aiming to achieve a similar effective refractive index with that of reduced thickness in crystalline state. The refractive indices of Si and SiO2 are 3.47 and 1.444 at 1550 nm.

For the MC, the width of the input and output waveguide is set to 1 μm to support both TE0 and TE1 modes. For the amorphous Sb_2_Se_3_ pattern, most of the input light goes through the MC without mode evolution, maintaining TE0 mode. After the Sb_2_Se_3_ pattern on top of the silicon is crystallized, the MC converts most of the input light (TE0) to TE1. Based on this requirement, the mode of the output light was monitored during optimization. The figure of merit of the MC is marked as *f* and the definition is as follows:

$$minimize\;\;\;f\left(z\right)=\left\{\begin{array}{cc} \left|T_{{\text{TE}}_{0}}-1\right|,\; & \;\text{if}\;\;z=z^{a}\\ \left|T_{{\text{TE}}_{1}}-1\right|,\; & \;\text{if}\;\;z=z^{c} \end{array}\right.$$

where $T_{{\text{TE}}_{0}}$ and $T_{{\text{TE}}_{1}}$ are the transmittance of TE0 and TE1 mode at the output port at amorphous and crystalline state, respectively, *n*_1_ and *n*_2_ are the refractive indices for the amorphous and crystalline state of Sb_2_Se_3_, respectively. During the iteration of optimization, the minimization of *f* was conducted for transforming most of the input from TE0 to TE1 at the crystalline state and maintaining TE0 mode at the amorphous state.

For the OS, the input port is at the center in the *y* direction, the output ports are symmetrically distributed and the distance is 2 μm. The figure of merit for optimization was set

$$minimize\;\;f\left(z\right)=\left\{\begin{array}{cc} \left|T_{\text{Port}1}-1\right|,\; & \;\text{if}\;\;z=z^{a}\\ \left|T_{\text{Port}2}-1\right|,\; & \;\text{if}\;\;z=z^{c} \end{array}\right.$$

where *T*_Port1_ and *T*_Port2_ are the transmittance for output ports at crystalline and amorphous states, respectively. To achieve the design of a reconfigurable OS, the *f* was minimized during the optimization.

Typically, the size and thickness of core material have a great impact on the performance of inverse-designed photonic devices. In our design, the pattern of Sb_2_Se_3_ was designed to achieve different functions. To achieve a fabrication error-insensitive design, the performance of the device is insensitive to the size and the thickness of the Sb_2_Se_3_ pattern on top of the silicon. The variation in size (Δ*s*) and thickness (Δ*t*) of the Sb_2_Se_3_ pattern are indicated in Figure 1(c). The simulation indicates that the OS exhibits a low insertion loss (IL) and high extinction ratio (ER) within a certain range (around ±10 nm in size) of fabrication error (see Figure 1(d) and (e)). Therefore, our proposed robust inverse design is a promising solution for designing reconfigurable photonic devices with the figure of merit of compact and especially fabrication error-tolerant.

The robust inverse design algorithm for reconfigurable photonic devices can be used for optimizing multifunctional devices for different refractive indices, which is of great significance for future multi-application photonic systems.

### 2.2 Design of mode converter

**Figure 2:**
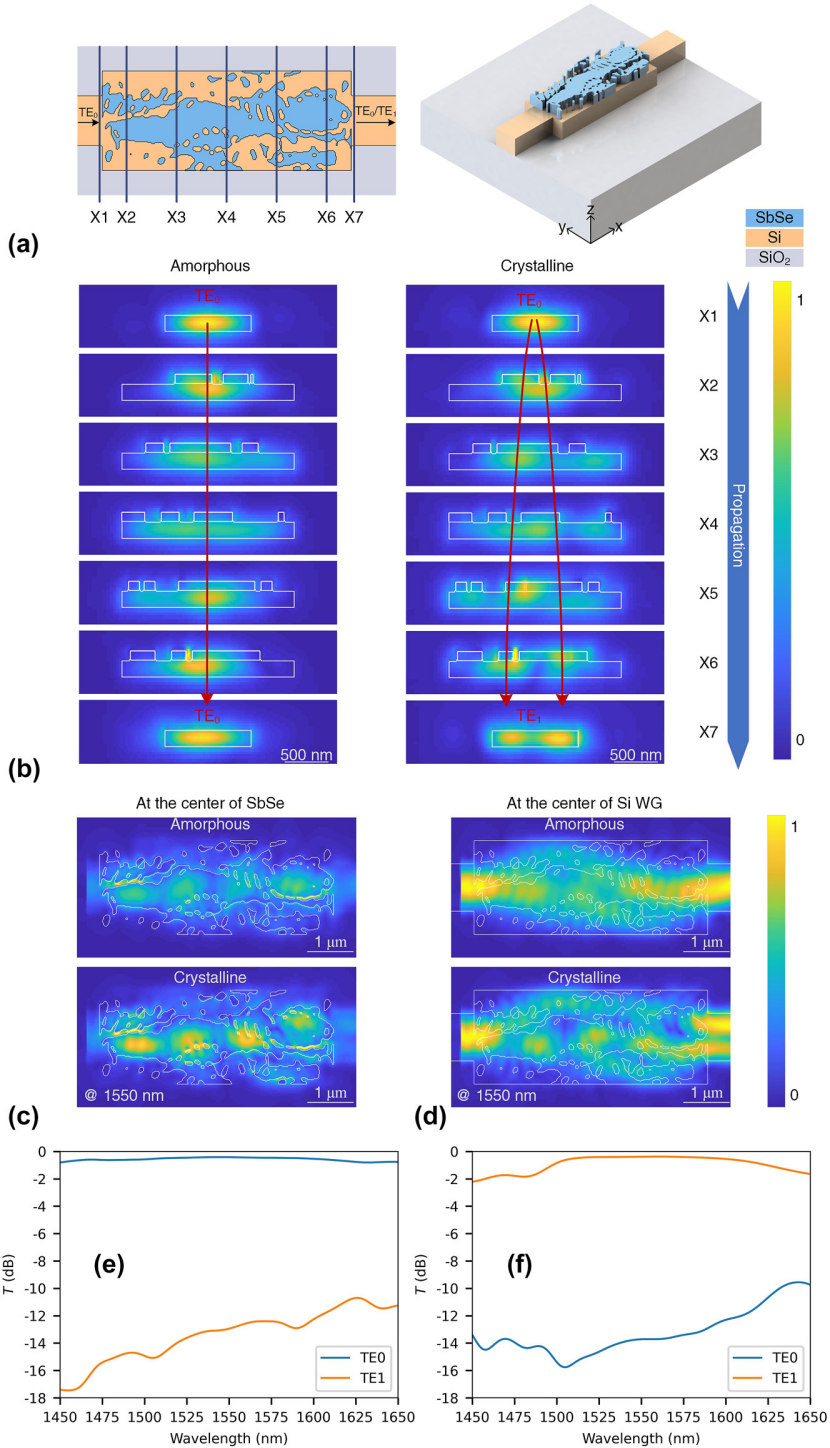
The schematic diagram and performance of the designed MC. (a) The layout and 3D image of the MC. The footprint of MC is 5 μm × 2 μm. X1 to X7 are different positions along the propagation direction of the devices. X1 is positioned in the input waveguide at a distance of 5 nm away from the input port of MC. X7 is located in the output waveguide, 5 nm away from the output port of MC. X2 to X6 is inside MC and has a distance of 0.5, 1.5, 2.5 3.5, 4.5 μm to the input port of MC. (b) The distribution of the electric field (*y*–*z* plane) of MC along the propagation direction. (c) The distribution of the electric field (*x*–*y* plane) at the center of the Sb_2_Se_3_ pattern. (d) The electric field (*x*–*y* plane) of MC at the center of the silicon waveguide. The simulated spectra at the amorphous state (e) and the crystalline state (f).

MC plays a pivotal role in achieving on-chip mode multiplexing, thus expanding the channel capacity [40]. By employing the robust inverse design algorithm, a thermally induced reconfigurable MC was designed, as depicted in Figure 2(a). The distribution of the electric field at different cross sections along the direction of light propagation is shown in Figure 2(b). When the Sb_2_Se_3_ structure on top of silicon is amorphous, although the light experiences certain perturbations while propagating in the MC, the output light remains in TE0 mode. In the crystalline state, the perturbations exhibit a relatively large magnitude due to the higher refractive index of crystalized Sb_2_Se_3_, resulting in the predominant conversion of light into TE1 mode.

The distribution of the electric field (*x*–*y* plane) at the center of the Sb_2_Se_3_ structure is shown in Figure 2(c). The crystalline Sb_2_Se_3_ has a more pronounced disturbance than that in the amorphous state, therefore achieving the mode converting from TE0 mode to TE1 mode. At the output port, the light intensity is close to the evanescent field at the input port. Therefore, the hybrid integration of Sb_2_Se_3_ offers a low insertion loss. The distribution of the electric field (*x*–*y* plane) at the center of the silicon waveguide is shown in Figure 2(d). A reconfigurable MC was achieved by converting TE0 to TE1 (maintaining TE0) at the crystalline (amorphous) state. The simulated spectra are shown in Figure 2(e) and (f), a broadband operating was achieved. The transmittance (*T*) serves as an indicator of the device’s insertion loss, whereby a 0 dB value signifies the absence of any insertion loss. At the amorphous state, the IL of TE0 mode is 0.42 dB and the corresponding ER is 12.5 dB at 1550 nm. The bandwidth, with an IL of <1 dB and ER of >10 dB, spans approximately 173 nm (ranging from 1450.0 nm to 1623.8 nm). At crystalline state, the IL is 0.39 dB with an ER of 13.3 dB at 1550 nm. The operating band, where IL is <1 dB and ER is >10 dB, ranges from 1498.1 nm to 1622.7 nm (bandwidth of nearly 124 nm). Therefore, by employing the robust inverse design, a compact and broadband operating MC was achieved, which is a promising candidate for a high-density integrated mode multiplexing system.

### 2.3 Design of optical switch

**Figure 3:**
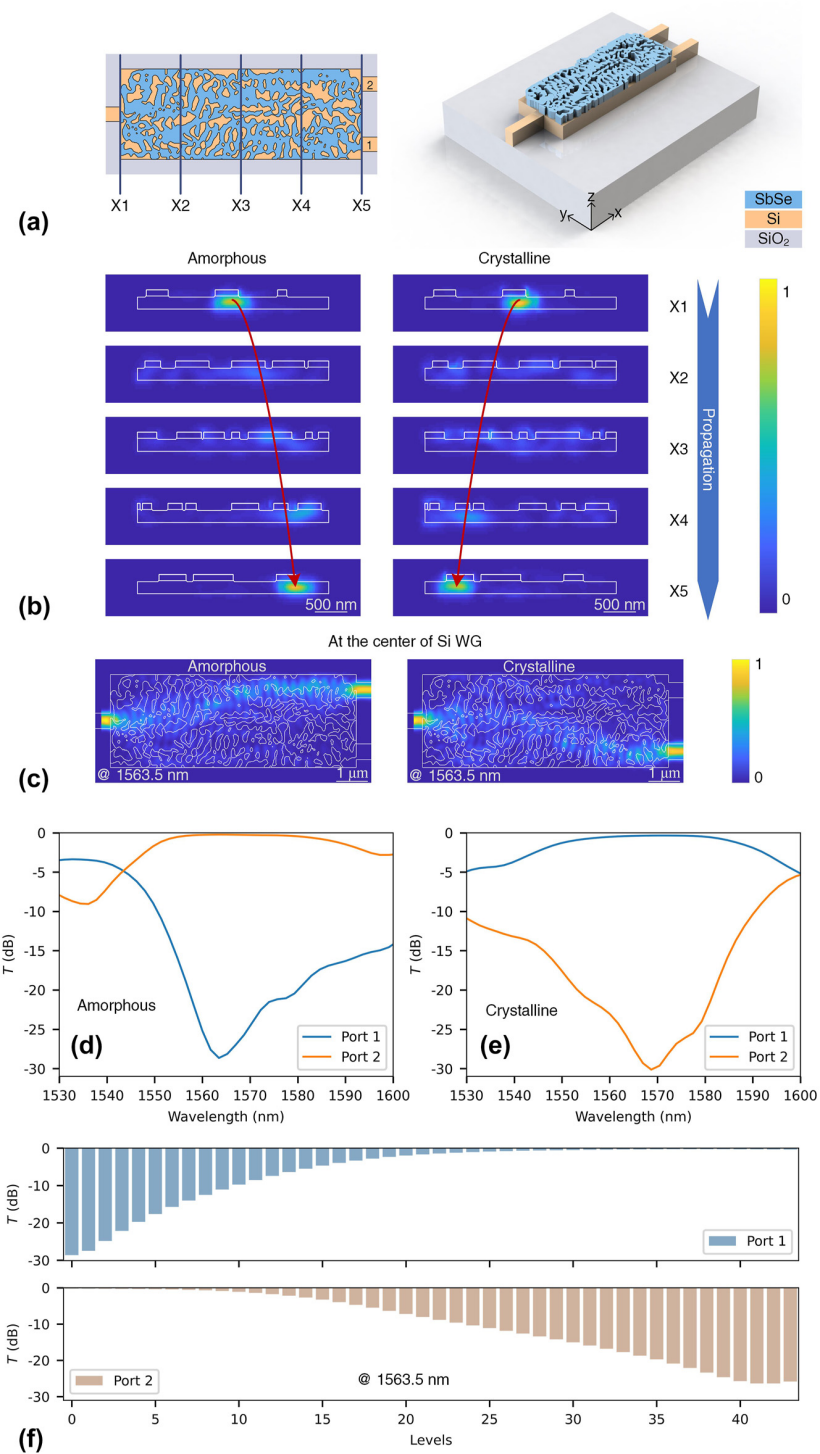
The structure diagram and performance of the OS. (a) The top view of the device and the 3D schematic diagram of the OS. X1 to X5 are different positions along the propagation direction of OS with a distance to the input port of 0, 2, 4, 6, and 8 μm, respectively. (b) The energy distribution (*y*–*z* plane) of different positions of OS at amorphous and crystalline states, respectively. (c) The energy flow (*x*–*y* plane) at the center of the silicon waveguide. The simulated spectra of amorphous (d) and crystalline (e) state. (f) The simulated multi-level switching of the OS at 1563.5 nm.

The OS is a key discrete device for optical systems such as optical computing [31]. A multilevel reconfigurable OS was designed by employing the robust inverse design, as the designed layout and 3D schematic image shown in Figure 3(a). The energy flow (*y*–*z* plane) in the cross section is shown in Figure 3(b), the light is gradually directed to port 1 (port 2) at the crystalline (amorphous) state. At the output port, the light is most confined in the silicon waveguide, achieving a low-loss design. Therefore, a phase transition-induced routing switch was achieved via the Sb_2_Se_3_/Si hybrid devices.

The energy flow at the center of the silicon waveguide is shown in Figure 3(c). When the Sb_2_Se_3_ is in its crystalline (amorphous) state, most of the input light is directed to port 1 (port 2). The simulated optical response at the amorphous state is shown in Figure 3(d). At 1563.5 nm, the device exhibits a lowest IL of approximately 0.23 dB with a high ER of 28.4 dB. The bandwidth of IL < 1 dB, ER > 10 dB is about 33 nm (from 1553.1 nm to 1586.4 nm). At crystalline state, the OS exhibits an IL of 0.41 dB with an ER of 25.6 dB (see Figure 3(e)). The operating waveband with IL < 1 dB and ER > 10 dB ranges from 1553.1 to 1584.6 nm, corresponding to a bandwidth of 31 nm. The OS may exhibit various states of splitting ratio. Therefore, the simulation of OS was carried out based on the interpolated refractive index corresponding to different states of Sb_2_Se_3_ [41]. By gradually increasing the crystallinity, a 41-level (>5-bits) multilevel switching was achieved in the simulation (see Figure 3(f)). Therefore, the OS has the potential to achieve multilevel switching as revealed by the simulation. Based on the Sb_2_Se_3_ and Si heterogeneous integration platform, a reconfigurable OS with IL < 0.45 dB and ER > 25 dB with crystallization-induced multilevel switching (>5 bits) was designed.

### 2.4 Device fabrication and measurement

**Figure 4:**
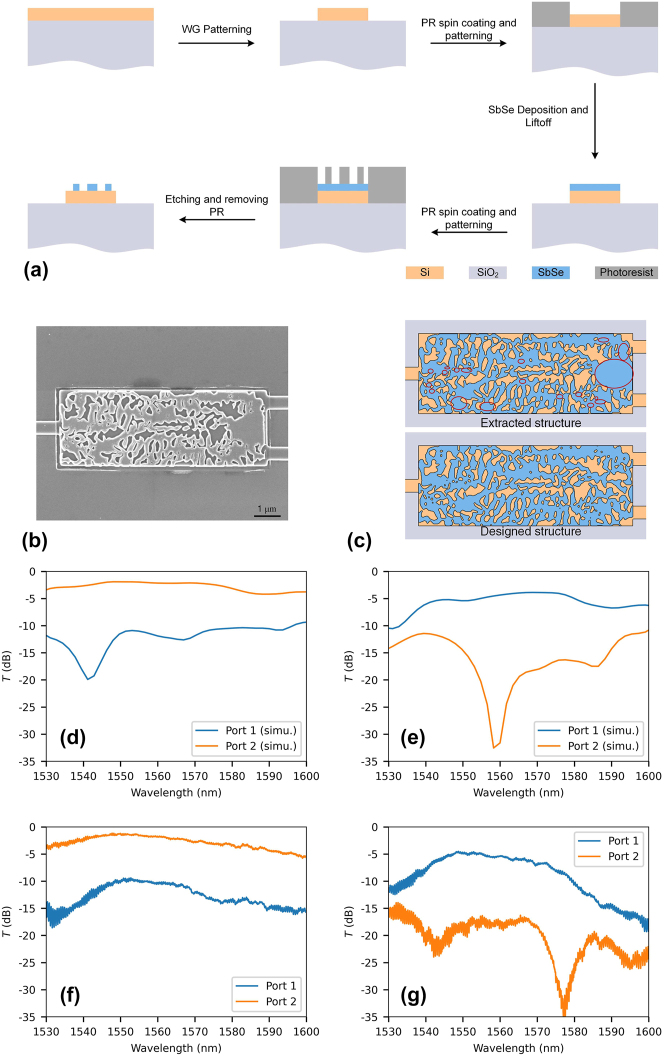
The fabrication and characterization of the fabrication error-tolerant OS. (a) The flow chart for device fabrication. (b) The scanning electron microscope (SEM) image of the fabricated device. (c) Comparison of the extracted pattern with the designed structure. The section highlighted within the red circle represents the missing pattern of the fabricated device. The simulated optical response of the extracted structure at amorphous (d) and crystalline (e) states. The measured spectra of the fabricated device at amorphous (f) and crystalline (g) states.

With the help of electron-beam lithography (EBL) and dry etching of silicon and Sb_2_Se_3_, for the first time, an inverse-designed, thermally induced routing switching OS was experimentally demonstrated. The OS was fabricated on a standard SOI (a layer of 220-nm silicon on top of 2 μm buried oxide), the fabrication flow chart is shown in Figure 4(a). Firstly, the silicon waveguide was defined by EBL, followed by RIE to etch it to a depth of 220 nm. Secondly, a layer of PMMA A4 was spin-coated onto the chip and subsequently patterned by EBL, thereby defining the window of Sb_2_Se_3_ deposition. Following thermal evaporation and lift-off processes, a 120-nm Sb_2_Se_3_ patch was fabricated onto the functional region. Thirdly, a layer of photoresist (APR 6200.13) was spin-coated onto the chip and then defined using EBL. Finally, the excess Sb_2_Se_3_ was subjected to etching, followed by the removal of the photoresist. To mitigate silicon damage, a CHF_3_-free dry etch recipe is employed. The dry etching process utilized an inductively coupled plasma etcher (Plasmapro 100, OXFORD) with an RF power of 200 W and ICP power of 300 W. The gas mixture consisting of 10 sccm of CH_4_ and 30 sccm of Ar was employed. The pressure of chamber is maintained at 5 mTorr, while the sample holder is kept at 20 °C. The temperature of the process following film deposition is lower than that required for inducing the crystallization of Sb_2_Se_3_. Consequently, the Sb_2_Se_3_ pattern remains in its amorphous state after fabrication, thereby facilitating convenient characterization of OS.

Leveraging the robust inverse design algorithm we proposed, the OS remains functional despite variations in size and the partial absence of the Sb_2_Se_3_ pattern. As the SEM image illustrated in Figure 4(b), some patterns of the fabricated device are lost owing to manufacturing errors, which is mainly attributed to occasional errors encountered during electron beam lithography (EBL). The comparison between the fabricated device and the designed device is shown in Figure 4(c), the lost structure is marked in the extracted structure. Despite these structural deficiencies, the OS still works at both states in the simulation (see Figure 4(d) and (e)). After fabrication, the OS was characterized using a home-built vertical-coupling platform as our previous work [42]. For a more comprehensive description of the measurement setup, refer to SI.2. The majority of light output was observed from port 2 (see Figure 4(f)). The measured spectra (*T*) is obtained by subtracting the measured spectrum to reference of grating coupler. At 1550 nm, the OS exhibits an IL of 1.28 dB and an ER of 8.79 dB. After annealing at 473 K for 10 min in the glove box, the beam-splitting ratio of the OS was altered as a result of Sb_2_Se_3_ crystallization, leading to the redirection of input light towards port 1 (see Figure 4(g)). The IL and ER of the OS are 4.98 dB and 14.43 dB, respectively. The measured optical response is similar to the simulated one, indicating a size variation insensitive design was achieved. Here, a crystallization-induced routing switching was demonstrated, which is a potential scheme compatible with electrical reprogramming utilizing a doped silicon heater or transparent electrode-based heater (ITO or graphene).

## 3 Conclusions

In this work, compact, fabrication error-tolerant, reconfigurable integrated photonic devices were developed by a robust inverse design method. Employing such an algorithm, a reconfigurable mode converter and a reconfigurable optical switch were designed with a structure of the hybrid integration of low-loss phase change material Sb_2_Se_3_ and silicon. The mode converter possesses a low IL (<1 dB) bandwidth of >100 nm. The optical switch exhibits an IL of <0.5 dB, corresponding to an ER of >25 dB and a multilevel switching of 41 levels (>5 bits). To the best of our knowledge, we, for the first time, experimentally demonstrated a crystallization-induced reconfigurable, Sb_2_Se_3_/Si hybrid integrated, compact optical switch, which exhibits a minimum IL of <1 dB, and minimum ER of >8.5 dB. These compact photonic devices employing robust inverse design are promising candidates for applications such as mode multiplex, optical routing, and optical computing, which will intriguing more designs of compact, multifunctional, and reconfigurable photonic devices for functional diversity optical chips.

## Supplementary Material

Supplementary Material Details

## References

[1] Pai S. (2023). Experimentally realized in situ backpropagation for deep learning in photonic neural networks. *Science*.

[2] Marpaung D., Yao J. P., Capmany J. (2019). Integrated microwave photonics. *Nat. Photonics*.

[3] Sun C. (2015). Single-chip microprocessor that communicates directly using light. *Nature*.

[4] Schaller R. R. (1997). Moore’s law: past, present and future. *IEEE Spectr.*.

[5] Molesky S., Lin Z., Piggott A. Y., Jin W., Vucković J., Rodriguez A. W. (2018). Inverse design in nanophotonics. *Nat. Photonics*.

[6] Piggott A. Y., Lu J., Lagoudakis K. G., Petykiewicz J., Babinec T. M., Vučković J. (2015). Inverse design and demonstration of a compact and broadband on-chip wavelength demultiplexer. *Nat. Photonics*.

[7] Piggott A. Y. (2020). Inverse-designed photonics for semiconductor foundries. *ACS Photonics*.

[8] Lu J., Vučković J. (2013). Nanophotonic computational design. *Opt. Express*.

[9] Wang K., Ren X., Chang W., Lu L., Liu D., Zhang M. (2020). Inverse design of digital nanophotonic devices using the adjoint method. *Photon. Res.*.

[10] Sapra N. V. (2020). On-chip integrated laser-driven particle accelerator. *Science*.

[11] Lin X. (2023). Compact mid-infrared chalcogenide glass photonic devices based on robust-inverse design. *Laser Photon. Rev.*.

[12] Cheng J., Zhang W., Gu W., Zhou H., Dong J., Zhang X. (2022). Photonic emulator for inverse design. *ACS Photonics*.

[13] Liu S. (2022). Thermo-optic phase shifters based on silicon-on-insulator platform: state-of-the-art and a review. *Front. Optoelectron.*.

[14] Youngblood N., Ocampo C. A. R., Pernice W. H. P., Bhaskaran H. (2023). Integrated optical memristors. *Nat. Photonics*.

[15] Zhang Y. (2019). Broadband transparent optical phase change materials for high-performance nonvolatile photonics. *Nat. Commun.*.

[16] Delaney M., Zeimpekis I., Lawson D., Hewak D. W., Muskens O. L. (2020). A new family of ultralow loss reversible phase-change materials for photonic integrated circuits: Sb_2_S_3_ and Sb_2_Se_3_. *Adv. Funct. Mater.*.

[17] Dong W. (2018). Wide bandgap phase change material tuned visible photonics. *Adv. Funct. Mater.*.

[18] Zhang H. (2019). Miniature multilevel optical memristive switch using phase change material. *ACS Photonics*.

[19] Zheng J. (2020). Nonvolatile electrically reconfigurable integrated photonic switch enabled by a silicon PIN diode heater. *Adv. Mater.*.

[20] Li W. (2022). Ultracompact high-extinction-ratio nonvolatile on-chip switches based on structured phase change materials. *Laser Photon. Rev.*.

[21] Zhang C. (2023). Nonvolatile multilevel switching of silicon photonic devices with In2O3/GST segmented structures. *Adv. Opt. Mater.*.

[22] Sun B. (2023). Integrated Bragg grating filters based on silicon-Sb_2_Se_3_ with non-volatile bandgap engineering capability. *Opt. Express*.

[23] Ríos C. (2022). Ultra-compact nonvolatile phase shifter based on electrically reprogrammable transparent phase change materials. *PhotoniX*.

[24] Chen R. (2023). Non-volatile electrically programmable integrated photonics with a 5-bit operation. *Nat. Commun.*.

[25] Yang X. (2023). Non-volatile optical switch element enabled by low-loss phase change material. *Adv. Funct. Mater.*.

[26] Wei M. (2023). Electrically programmable phase-change photonic memory for optical neural networks with nanoseconds *in situ* training capability. *Adv. Photon.*.

[27] Wu C., Yu H., Lee S., Peng R., Takeuchi I., Li M. (2021). Programmable phase-change metasurfaces on waveguides for multimode photonic convolutional neural network. *Nat. Commun.*.

[28] Ríos C. (2015). Integrated all-photonic non-volatile multi-level memory. *Nat. Photonics*.

[29] Feldmann J., Youngblood N., Li X., Wright C. D., Bhaskaran H., Pernice W. H. P. (2020). Integrated 256 cell photonic phase-change memory with 512-bit capacity. *IEEE J. Sel. Top. Quantum Electron.*.

[30] Feldmann J., Youngblood N., Wright C. D., Bhaskaran H., Pernice W. H. P. (2019). All-optical spiking neurosynaptic networks with self-learning capabilities. *Nature*.

[31] Feldmann J. (2021). Parallel convolutional processing using an integrated photonic tensor core. *Nature*.

[32] Brückerhoff-Plückelmann F. (2022). Broadband photonic tensor core with integrated ultra-low crosstalk wavelength multiplexers. *Nanophotonics*.

[33] Delaney M. (2021). Nonvolatile programmable silicon photonics using an ultralow-loss Sb_2_Se_3_ phase change material. *Sci. Adv.*.

[34] Yuan H., Wang Z., Peng Z., Wu J., Yang J. (2023). Ultra-compact and non volatile nanophotonic neural networks. *Adv. Opt. Mater.*.

[35] Yang S., Huang Y., He P., Liu D., Zhang M. (2023). Ultracompact programmable inverse-designed nanophotonic devices based on digital subwavelength structures. *Appl. Opt.*.

[36] Ma H., Yang J., Huang J., Zhang Z., Zhang K. (2021). Inverse-designed single-mode and multi-mode nanophotonic waveguide switches based on hybrid silicon-Ge2Sb2Te5 platform. *Results Phys.*.

[37] Ma H. (2022). Inverse design of nonvolatile reconfigurable mode generator and optical circulator based on a novel concept of a fully-digitized module. *J. Lightwave Technol.*.

[38] Jiao Z., Wu C., Yu H., Takeuchi I., Ríos C., Li M. (2023). Programmable mode/wavelength demultiplexer using inverse-designed low-loss phase-change material. *CLEO 2023*.

[39] Lei K. (2022). Magnetron-sputtered and thermal-evaporated low-loss Sb-Se phase-change films in non-volatile integrated photonics. *Opt. Mater. Express*.

[40] Li C., Liu D., Dai D. (2019). Multimode silicon photonics. *Nanophotonics*.

[41] Wright C. D., Liu Y., Kohary K. I., Aziz M. M., Hicken R. J. (2011). Arithmetic and biologically-inspired computing using phase-change materials. *Adv. Mater.*.

[42] Xie X. (2023). Harnessing anti-parity-time phase transition in coupled topological photonic valley waveguides. *Adv. Funct. Mater.*.

