# Supplementary material for: Inverse design of compact nonvolatile reconfigurable silicon photonic devices with phase-change materials
Source: Nanophotonics. 2024 Jan 15;13(12):2183–92. doi: 10.1515/nanoph-2023-0637 (PMC11502029; doi:10.1515/nanoph-2023-0637)
Supplement: Supplementary file 1 — Supplementary Material Details [file j_nanoph-2023-0637_suppl_001.pdf]

Supplementary Information

Inverse design of compact nonvolatile reconfigurable silicon photonic devices with phase-change materials

Maoliang Wei†, Xiaobin Lin†, Kai Xu, Yingchun Wu, Chi Wang, Zijia Wang, Kunhao Lei, Kangjian Bao, Junying Li, Lan Li, Erping Li, and Hongtao Lin*

***Corresponding author: Hongtao Lin**, The State Key Lab of Brain-Machine Intelligence, Key Laboratory of Micro-Nano Electronics and Smart System of Zhejiang Province, College of Information Science and Electronic Engineering, Zhejiang University, Hangzhou 310027, China; hometown@zju.edu.cn; 0000-0001-7432-3644

Maoliang Wei, Xiaobin Lin, Kai Xu, Chi Wang, Zijia Wang, Kunhao Lei, and Erping Li: The State Key Lab of Brain-Machine Intelligence, Key Laboratory of Micro-Nano Electronics and Smart System of Zhejiang Province, College of Information Science and Electronic Engineering, Zhejiang University, Hangzhou 310027, China

Yingchun Wu, Kangjian Bao, and Lan Li: Key Laboratory of 3D Micro/Nano Fabrication and Characterization of Zhejiang Province, School of Engineering, Westlake University, Hangzhou, Zhejiang 310030, China and Institute of Advanced Technology, Westlake Institute for Advanced Study, Hangzhou, Zhejiang 310024, China

Junying Li: Hangzhou Institute for Advanced Study, University of Chinese Academy of Sciences, Hangzhou 310024, China

†These authors contributed equally.

1. The principle of algorithm

The operation of the algorithm includes three parts (Adjoint Optimization, Filter and Projection, and Perturbation). During Adjoint Optimization, the design objective (figure of merit, FOM) is usually expressed as a function of the field, which serves to estimate the level of agreement between the design and the target fields. This objective function can be expressed as the property of the field. Based on the optimization of this objective function, the desired device can be optimized. Therefore, the design of the device is transformed into a mathematical optimization problem. For a passive device, the FOM can be expressed as

$$\begin{aligned} &\text{minimize} \quad f(\boldsymbol{z}) \\ &\text{subject to} \quad A(\theta)\boldsymbol{z} = \boldsymbol{b}(\theta) \end{aligned}$$

where $\boldsymbol{z}$ is the electric field, θ is the parameters which we are aimed to design. For a reconfigurable inverse-designed device (two distinct states in our design), the electric field subject to the constraint conditions can be expressed as

$$A^c(\theta)\boldsymbol{z}^c = \boldsymbol{b}^c(\theta)$$

$$A^a(\theta)\boldsymbol{z}^a = \boldsymbol{b}^a(\theta)$$

where a, and c are two distinct states of the device (a (c) for amorphous (crystalline) state, respectively). Therefore, the FOM can be regarded as

$$\begin{aligned} &\text{minimize} \quad f(\boldsymbol{z}^c, \boldsymbol{z}^a) \\ &\text{subject to} \quad A^i(\theta)\boldsymbol{z}^i = \boldsymbol{b}^i(\theta) \quad i = c, a \end{aligned}$$

Among them, the simple form of each parameter is as follows

$$A(\theta) = \begin{bmatrix} A^a(\theta) & 0 \\ 0 & A^c(\theta) \end{bmatrix}$$

$$b(\theta) = \begin{bmatrix} b^a(\theta) \\ b^c(\theta) \end{bmatrix}, \quad \boldsymbol{z} = \begin{bmatrix} \boldsymbol{z}^a \\ \boldsymbol{z}^c \end{bmatrix}$$

Therefore, the derivative of the objective function (f) with respect to the design parameters (θ) is

$$\frac{\partial}{\partial \theta_i} f\left(A(\theta)^{-1} b(\theta)\right) = \left(A(\theta)^{-T} \left(\nabla f\left(\boldsymbol{z}\right)\right)\right)^T A_i \boldsymbol{z}$$

The gradient of the objective function (f) with respect to the variable can be calculated by solving only two systems of linear equations: $A(\theta)$ and $A(\theta)^T$, which is referred to as the adjoint method.

Based on the above-mentioned Adjoint Optimization, the parameters can be optimized. However, the optimized parameters are continuously varied. Therefore, the Filter and Projection are introduced to execute binarization. Furthermore, to achieve a manufacturing error-tolerant design, the Perturbation is introduced, and the FOM can be expressed as

$$\min_{\theta} \quad f(z) = \begin{cases} f_0(z) & \beta \le \beta_0 \\ f_0(z) + E_{\xi}\left[\left(f^*(z^{\xi}) - f_0(z)\right)^2\right] & \beta > \beta_0 \end{cases}$$

Based on this robust inverse design algorithm, compact reconfigurable devices can be designed.

2. The measurement setup

The device under test (DUT) was measured by employing a home-built vertical-coupling platform. The spectral scanning measurement is conducted by employing a computer to control the laser (Santec TSL-550) and detector (MPM-210). The laser-generated optical signal (ranging from 1480 to 1630 nm) was transmitted through the polarization controller, coupled to the DUT via the coupling system, and ultimately collected by a detector.
